# Alveolar-like Macrophages Attenuate Respiratory Syncytial Virus Infection

**DOI:** 10.3390/v13101960

**Published:** 2021-09-29

**Authors:** Bárbara N. Porto, Michael L. Litvack, Yuchen Cen, Irene Lok, Sheena Bouch, Michael J. Norris, Wenming Duan, Cameron Ackerley, Martin Post, Theo J. Moraes

**Affiliations:** 1 Program in Translational Medicine, Hospital for Sick Children, Toronto, ON M5G 0A4, Canada; bnporto@hotmail.com (B.N.P.); michael.litvack@sickkids.ca (M.L.L.); samcen2003@yahoo.ca (Y.C.); i.m.lok@amsterdamumc.nl (I.L.); sheena.bouch@sickkids.ca (S.B.); mnorris@lji.org (M.J.N.); wenming.duan@sickkids.ca (W.D.); cameron.ackerley@sickkids.ca (C.A.); martin.post@sickkids.ca (M.P.); 2 Department of Laboratory Medicine and Pathobiology, University of Toronto, Toronto, ON M5S 1A8, Canada; 3 Neonatal Intensive Care Unit, Emma Children’s Hospital, Amsterdam UMC, University of Amsterdam, 1105 AZ Amsterdam, The Netherlands; 4 Center for Infectious Disease and Vaccine Research, La Jolla Institute for Immunology, La Jolla, CA 92037, USA; 5 Division of Respiratory Medicine, Department of Pediatrics, Hospital for Sick Children, Toronto, ON M5G 1X8, Canada

**Keywords:** Respiratory Syncytial Virus, alveolar macrophages, stem cells, respiratory infection

## Abstract

Respiratory Syncytial Virus (RSV) is the leading cause of acute lower respiratory infections in young children and infection has been linked to the development of persistent lung disease in the form of wheezing and asthma. Despite substantial research efforts, there are no RSV vaccines currently available and an effective monoclonal antibody targeting the RSV fusion protein (palivizumab) is of limited general use given the associated expense. Therefore, the development of novel approaches to prevent RSV infection is highly desirable to improve pediatric health globally. We have developed a method to generate alveolar-like macrophages (ALMs) from pluripotent stem cells. These ALMs have shown potential to promote airway innate immunity and tissue repair and so we hypothesized that ALMs could be used as a strategy to prevent RSV infection. Here, we demonstrate that ALMs are not productively infected by RSV and prevent the infection of epithelial cells. Prevention of epithelial infection was mediated by two different mechanisms: phagocytosis of RSV particles and release of an antiviral soluble factor different from type I interferon. Furthermore, intratracheal administration of ALMs protected mice from subsequent virus-induced weight loss and decreased lung viral titres and inflammation, indicating that ALMs can impair the pathogenesis of RSV infection. Our results support a prophylactic role for ALMs in the setting of RSV infection and warrant further studies on stem cell-derived ALMs as a novel cell-based therapy for pulmonary viral infections.

## 1. Introduction

Respiratory Syncytial Virus (RSV) is the leading global infectious cause of acute lower respiratory infections in children under 2 years of age [1]. It is estimated that RSV is responsible for more than 3 million hospital admissions and approximately 118,000 deaths every year worldwide [2]. In addition to acute disease, there is evidence suggesting that RSV infection in childhood may trigger persistent or recurrent wheezing and asthma in later life [3,4]. Despite extensive research efforts, there is currently no effective treatment or vaccine, highlighting the need for development of novel therapies and/or ways to prevent RSV infection.

Alveolar macrophages (AMs) are strategically located in the terminal airways and survey the lung against inhaled microbes [5,6]. AMs recognize pathogen-associated molecular patterns via Toll-like receptors (TLR) triggering an immune response against the invading microbes via the release of inflammatory factors and phagocytosis of the pathogens [5]. AMs have been identified as protective cells against respiratory viral infections. Mice with constitutive deficiencies in macrophage function have increased viral load and viral-induced airway occlusion after RSV infection [7]. Furthermore, AM depletion dampens the innate immune response to RSV, enhances viral replication and pathological score in the lungs [8,9].

Recently, we generated alveolar-like macrophages (ALMs) from pluripotent stem cells that better represent the fetal and embryonic origin of the native AM than blood-derived macrophages used in previous studies [10]. These ALMs have shown potential to promote enhanced airway innate immunity and tissue repair [11]. Due to their derivation from stem cells, ALMs have substantial proliferative capacity and can be scaled for corrective use with little change in function and phenotype. We, therefore, hypothesized that ALMs could contribute to enhanced clearance of RSV by bolstering the native innate immune functions of the lungs. In this study, we used several in vitro techniques to evaluate changes in infectivity of RSV in the presence of ALMs and then employed an animal model of RSV infection to evaluate the prophylactic potential of ALMs. We also explored the potential mechanism of interaction between ALMs and RSV. Taken together, our data show that ALMs reduce the infectivity of RSV in vitro and in vivo. These findings endorse the idea that an ALM cell therapy offers a novel means to enhance immunity to respiratory viruses in a broad, non-specific manner.

## 2. Materials and Methods

### 2.1. Reagents

Eagle’s Minimum Essential Medium (EMEM, cat 320-006-CL), Dulbecco’s Modified Eagle’s Medium (DMEM, cat 219-010-XK) and phosphate-buffered saline (PBS, cat 311-011-CL) were purchased from Wisent Inc. Heat-inactivated FBS (cat 10082147) and αMEM (cat 12571063) were from Gibco^TM^. Anti-RSV F protein antibody (clone 133-1H) was from EMD-Millipore (cat MAB8262) and anti-F4/80 (clone BM8) was from eBioscience (cat MA5-16624). Cytochalasin D from *Zygosporium mansonii* was from Sigma (cat C8273). Poly I:C (HMW) was from InvivoGen (cat tlrl-pic). Hoechst 33342 was from Invitrogen^TM^ (cat 62249).

### 2.2. Cell Lines and Viruses

HEp-2 cells (ATCC CCL-23) were maintained in EMEM supplemented with 10% (*v/v*) heat-inactivated fetal bovine serum (FBS) and 1% (*v/v*) penicillin-streptomycin solution. NIH/3T3 mouse fibroblasts (ATCC CRL-1658) were maintained in DMEM supplemented with 10% (*v/v*) FBS and 1% (*v/v*) penicillin-streptomycin solution. The cells were grown in a humidified incubator at 37 °C with 5% CO2. Sf9 cells (Invitrogen^TM^, cat 11496015) were maintained at 27 °C in Grace’s supplemented media (Invitrogen^TM^, cat 11605094) containing 10% (*v/v*) heat-inactivated FBS.

The A2 strain of RSV was purchased from ATCC (VR-1540). The recombinant strain of RSV expressing green fluorescent protein (rgRSV224; RSV-GFP) was kindly provided by Dr. M. E. Peeples (Children’s Research Institute, Columbus, OH, USA) and Dr. P. L. Collins (National Institutes of Health, Bethesda, MD, USA). RSV stocks were produced in HEp-2 cells as previously described [12]. Briefly, 80–90% confluent HEp-2 cells were incubated with RSV in serum-free EMEM for 2 to 4 h while rotating flasks every 30 min. Then, EMEM supplemented with 6% FBS was added to flasks and cells incubated for 3 to 4 days. By day 4, usually 50% of cells were detached and virus was harvested and purified. For purification, infected HEp-2 cells were scraped and collected into Falcon tubes and centrifuged at 800× *g* for 10 min. The cell pellet was subjected to two freeze–thaw cycles and sonication for 20 sec on ice. Then, the cell pellet was discarded and the supernatants were ultra-centrifuged at 110,000× *g* for 45 min over a sucrose gradient (30% sucrose in 0.1 M sodium chloride, 0.01 M Tris-HCl, 0.001 M EDTA, 1 M urea, pH 7.5). After that, a virus pellet was visible and the supernatant was decanted. RSV pellet was resuspended in 100 μL of PBS per T-150 flask and stored in −80 °C.

### 2.3. Alveolar-like Macrophages (ALM)

Alveolar-like macrophages were generated as described previously [11]. Briefly, ALMs were derived from dsRed-expressing mouse embryonic stem cells [13] and cultured in serum-free factor defined conditions through a directed differentiation from pluripotency to hemogenic mesoderm followed by primitive hematopoiesis to generate primitive myb-independent macrophages. These macrophages were then conditioned in media containing 10 ng/mL of recombinant mouse M-CSF (R&D Systems cat 416-ML/CF) and 20 ng/mL of recombinant GM-CSF (R&D Systems cat 415-ML/CF) to support macrophage growth in alveolar-like conditions. ALMs were sorted for their dual expression of F4/80 and CD11c using FACS analysis and they were verified to express CD45, CD11b, CD11c and F4/80, with no expression of MHC class II [11]. Macrophages were maintained under indicated conditions.

### 2.4. Peripheral Blood Mononuclear Cells (PBMC)

PBMCs were isolated from healthy volunteer donors who had signed an informed consent form. A total of 20 mL of whole blood was collected and subjected to a Histopaque-1077 (Sigma-Aldrich, cat 10771) gradient centrifugation for 30 min. PBMCs were seeded at 2 × 10^5^/well in EMEM supplemented with 10% (*v/v*) heat-inactivated FBS overnight. Non-adherent cells were then removed by washing, and adherent cells were exposed to RSV-GFP (MOI 1) for 90 min. Then, unbound virus was removed and fresh EMEM containing 10% (*v/v*) FBS was added to the cells. RSV-GFP-infected cells were imaged at 24 h intervals for 72 h on an inverted epifluorescence microscope (Nikon TE-2000), using a Hamamatsu C4742-12AG camera and Perkin Elmer Volocity software. The total fluorescence intensity was determined from 4 random fields per well (4× magnification) using Perkin Elmer Volocity software, as previously described [14].

### 2.5. Primary Alveolar Macrophages (PAM)

PAMs were collected by bronchoalveolar lavage (BAL) by flushing the lungs of BALB/c mice three times with 1 mL of chilled EMEM supplemented with EDTA (5 mM). The lavage was repeated twice and PAMs from several mice were pooled. Cells were seeded at 2 × 10^5^/well in EMEM supplemented with 10% (*v/v*) FBS overnight. Non-adherent cells were then removed by washing, and adherent cells were exposed to RSV-GFP (MOI 1) for 90 min. Then, unbound virus was removed and fresh EMEM containing 10% (*v/v*) FBS was added to the cells. RSV-GFP-infected cells were imaged at 24 h intervals for 72 h on an inverted epifluorescence microscope (Nikon TE-2000), using a Hamamatsu C4742-12AG camera and Perkin Elmer Volocity software. The total fluorescence intensity was determined from 4 random fields per well (4× magnification) using Perkin Elmer Volocity software, as previously described [14].

### 2.6. Human Umbilical Cord Blood-Mesenchymal Stromal Cells (hUCB-MSC)

hUCB-MSC were isolated as previously described [15]. Briefly, hUCB-MSCs were isolated from the umbilical cord of healthy, term pregnancies after parental consent and were kindly provided by Dr. Bernard Thébaud. After delivery, the cord was dissected from the placenta, milked to remove remaining intravascular blood and placed in a storage solution containing 30% (*v/v*) citrate phosphate dextrose adenine anti-coagulant (CPDA-1) in PBS. After disinfection, multiple incisions were made into the epithelium of the cord. Sterile cord tape (Ethicon, cat U10T) was used to ligate the cord vessels prior to placing the tissue in Dulbecco’s PBS (D-PBS) supplemented with Ca2+, Mg2+, glucose and pyruvate as well as with 750 U/mL collagenase (Worthington, cat LS004152) and 500 U/mL hyaluronidase (Worthington, cat LS005477). Following digestion at 38 °C for 4 h, 2000 BAEE-U/mL trypsin (Sigma-Aldrich, cat T4799) and 3 mM EDTA were added and allowed to incubate for another 30 min at 38 °C. The dissociation process was stopped by placing the mixture on ice and adding FBS to a final concentration of 10% (*v/v*). Remaining undigested tissue was removed by utilizing 40 µm cell strainers. Cells were rinsed in PBS and seeded in MEM, alpha modification (αMEM) containing 20% (*v/v*) FBS in tissue culture flasks. Culture was maintained in a 5% CO_2_, 5% O_2_ humidified atmosphere with media changes every 3rd day. Upon reaching 80–90% confluence, cells were lifted using recombinant trypsin (Thermo Fisher Scientific, Waltham, Massachusetts, USA, cat 12563011) and re-seeded as passage 1 (P1) MSCs at 5000 cells per cm^2^.

### 2.7. RSV Infection of HEp-2 Cells and ALM

HEp-2 cells have been widely used in the study of respiratory viruses as they are readily infected; in this work, they were used as a read out of RSV infection and replication. HEp-2 cells (1 × 10^4^/well) or ALMs (1 × 10^5^/well) were plated in black, flat, clear-bottom 96-well plates and incubated overnight at 37 °C with 5% CO_2_. Afterwards, cells were washed with serum-free EMEM and incubated with RSV-GFP (MOI 1) for 90 min. Then, unbound virus was removed and fresh EMEM containing 10% (*v/v*) FBS was added to the cells. RSV-GFP-infected cells were imaged at 24 h intervals for 72 h on an inverted epifluorescence microscope (Nikon TE-2000), using a Hamamatsu C4742-12AG camera and Perkin Elmer Volocity software. The total fluorescence intensity was determined from 4 random fields per well (4× magnification) using Perkin Elmer Volocity software, as previously described [14].

### 2.8. Conditioned Media Assay

ALMs (1 × 10^6^/mL) were incubated with RSV-GFP (MOI 1) for 1 h at 37 °C with 5% CO_2_. Afterwards, cells were spun down at 800 *g* for 10 min at 4 °C. ALM supernatants were UV-inactivated at 1200 μJ/cm^2^ for 30 min using a UV Stratalinker 2400 (Stratagene). Then, the UV-inactivated conditioned media was added to HEp-2 cells at a ratio of 1:1 with EMEM and subsequently infected with RSV-GFP (MOI 1) for 48 h at 37 °C in 5% CO_2_. After this period, HEp-2 cells were imaged on an inverted epifluorescence microscope as described above.

### 2.9. Phagocytosis Inhibition Assays

To evaluate the role of RSV phagocytosis on ALM antiviral effect, ALMs (1 × 10^6^/600 μL) were pretreated with cytochalasin D (10 μM) for 1 h at 37 °C in 5% CO_2_. Afterwards, ALM were stimulated with RSV-GFP (MOI 1) for 1 h and centrifuged at 800× *g* for 10 min at 4 °C to pellet the cells. HEp-2 cells (1 × 10^4^/well) were then stimulated with ALM conditioned media (200 μL) for 48 h at 37 °C. After this period, HEp-2 cells were imaged on an inverted epifluorescence microscope as described above. Alternatively, ALMs were incubated at 4 °C for 1 h and subsequently exposed to RSV (MOI 1) at 4 °C in a thermomixer with gentle agitation for another 1 h. After this period, cells were centrifuged at 800× *g* for 10 min at 4 °C and the supernatants were immediately used for RSV titration in a plaque assay as described below.

### 2.10. RSV Plaque Assay

To determine the viral titre, RSV-infected HEp-2 cells were harvested, submitted to 2 cycles of freeze–thaw and then inoculated onto HEp-2 cell monolayers in 6-well plates for 2 h at 37 °C. Cells were then overlaid with 1 mL of 1% agarose in DMEM and incubated for 5 days at 37 °C with 5% CO_2_. Afterwards, cells were fixed with formalin and lysis plate titration was performed using 0.05% (*v/v*) neutral red and viral titre was expressed as plaque forming units (PFU).

### 2.11. Immunofluorescence

ALM or HEp-2 cells were cytospun at 1000× *g* for 1 min onto a glass slide. Cells were fixed with 4% (*v/v*) PFA for 20 min and subsequently permeabilized with 0.3% (*v/v*) Triton-X 100 for 10 min. Afterwards, cells were stained with anti-RSV F protein (1:250) and anti-F4/80 (1:100) antibodies for 1 h, followed by Hoechst 33342 (1:4000) staining. Images were taken with a Leica DMi8 confocal microscope and analyzed using Improvision Volocity software with minor brightness and contrast adjustment not exceeding 20% of original values.

### 2.12. Transmission Electron Microscopy

Cells examined by electron microscopy were fixed in 2.5% (*w/v*) glutaraldehyde in 0.1 M phosphate buffer at pH 7.4 followed by 1% (wt/v) osmium tetroxide. Then, samples were dehydrated and embedded in Araldite-Epon resin (EMS, cat 13940). Ultrathin sections were prepared and stained in uranyl acetate and lead citrate before viewing. All samples were examined on a JEOL JEM 1011 transmission electron microscope (JEOL USA, Peabody, MA).

### 2.13. IFN-β Measurements

ALMs (1 × 10^6^/mL) were stimulated with RSV (MOI 1) or mock-stimulated for 1, 4 or 24 h at 37 °C under 5% CO_2_. Alternatively, ALMs were stimulated with TLR3 ligand Poly I:C (10 and 20 μg/mL) for 24 h at 37 °C under 5% CO_2_. Afterwards, supernatants were collected, and IFN-β concentrations were determined using ELISA (R&D Systems, cat MIFNB0), following the manufacturer’s instructions.

### 2.14. ALM Conditioned Media Preparation for Proteomic Analysis

After incubating ALMs with mock, RSV-GFP (MOI 1) or UV-RSV-GFP (MOI 1) for 4 h at 37 °C with 5% CO_2_, cells were centrifuged at 1000× *g* for 10 min and the supernatants were collected. ALMs conditioned media were concentrated using an Amicon Ultra-15 centrifugal filter (3 kDa MWCO) and centrifuged at 3500 rpm for 30 min intervals. The protein concentration was then determined and the solution was lyophilized.

### 2.15. Proteomic Analysis

Proteomic work was performed with the SPARC BioCentre at SickKids. Briefly, ALM conditioned media was reduced, alkylated, digested, and TMT labelled according to manufacturer’s directions (Thermo Fisher TMT 10 Plex). Labelled peptides from all samples were combined and lyophilized. Peptides were then fractionated into 8 fractions using the Pierce High pH Reversed-Phase Peptide Fractionation Kit (Thermo Fisher) as per manufacturer’s directions. Samples were analyzed on an Orbitrap analyzer (Q-Exactive, Thermo Fisher) outfitted with a nanospray source and EASY-nLC nano-LC system (Thermo Fisher). Lyophilized peptide mixtures were dissolved in 0.1% formic acid and loaded onto a 75 μm × 50 cm PepMax RSLC EASY-Spray column filled with 2 μM C18 beads (Thermo Fisher) at a pressure of 800 Bar. Peptides were eluted over 240 min at a rate of 250 nL/min using a gradient set up as 0–40% gradient of Buffer A (0.1% Formic acid; and Buffer B, 0.1% Formic Acid in 80% acetonitrile). Peptides were introduced by nano-electrospray into the Q-Exactive mass spectrometer (Thermo Fisher). The instrument method consisted of one MS full scan (525–1600 m/z) in the Orbitrap mass analyzer with an automatic gain control (AGC) target of 1e6, maximum ion injection time of 120 ms and a resolution of 35,000 followed by 15 data-dependent MS/MS scans with a resolution of 35,000, an AGC target of 5e5, maximum ion time of 100 ms, and one microscan. The intensity threshold to trigger a MS/MS scan was set to an underfill ratio of 1.0%. Fragmentation occurred in the HCD trap with normalized collision energy set to 29. The dynamic exclusion was applied using a setting of 50 s.

For the database searching, tandem mass spectra were extracted, charge state deconvoluted and deisotoped by Xcalibur version 2.2. All MS/MS samples were analyzed using Sequest (Thermo Fisher Scientific; version 1.4.1.14) and X! Tandem (The GPM, thegpm.org; version CYCLONE (2010.12.01.1)). Sequest was set up to search Human-Uniprot-Reviewed-8 August 2016.fasta (42118 entries) assuming the digestion enzyme trypsin. X! Tandem was set up to search a subset of the Human-Uniprot-Reviewed-8 August 2016database (84330 entries) also assuming trypsin. Sequest and X! Tandem were searched with a fragment ion mass tolerance of 0.020 Da and a parent ion tolerance of 10.0 PPM. Carbamidomethyl of cysteine and TMT6plex of lysine and the n-terminus were specified in Sequest and X! Tandem as fixed modifications. Deamidated of asparagine and glutamine and oxidation of methionine were specified in Sequest as variable modifications. Glu->pyro-Glu of the n-terminus, ammonia-loss of the n-terminus, gln->pyro-Glu of the n-terminus, deamidated of asparagine and glutamine and oxidation of methionine were specified in X! Tandem as variable modifications.

Criteria for protein identification: Scaffold (version Scaffold_4.6.2, Proteome Software Inc.) was used to validate MS/MS based peptide and protein identifications. Peptide identifications were accepted if they could be established at greater than 95.0% probability by the Scaffold Local FDR algorithm. Protein identifications were accepted if they could be established at greater than 95.0% probability and contained at least 2 identified peptides. Protein probabilities were assigned by the Protein Prophet Algorithm [16]. Proteins that contained similar peptides and could not be differentiated based on MS/MS analysis alone were grouped to satisfy the principles of parsimony.

### 2.16. Animals and RSV Infection

BALB/c mice (68 weeks old) were housed at the Animal Facility of SickKids Research Institute. Animal procedures were approved by the Animal Use Committee of SickKids Research Institute (AUP # 1000034753). On day −2, mice were anesthetized with 5% isoflurane and 1 × 10^6^ ALMs or fibroblasts, as a cellular control, were instilled intratracheally. On day 0, mice were anesthetized and infected intranasally with 5 × 10^6^ PFU of RSV A2 strain. All animals were weighed daily at the same time. Tissue collection and data analysis was performed 4 days post-infection. Sample sizes are indicated in the figure legends.

### 2.17. Bronchoalveolar Lavage (BAL) Fluid Collection

Mice were euthanized with an overdose of ketamine and xylazine and the tracheas were cannulated. The lungs were lavaged twice with chilled PBS (1 mL). BAL was centrifuged and the supernatants collected for cytokine analysis using the Bio-Plex Pro Mouse Cytokine Singleplex Sets combined (KC, MCP-1, IL-6, TNF-α, MIP-1α, IFN-γ, IL-1β, IL-17A, IL-12p40, GM-CSF, Eotaxin, IL-10, IL-4, IL-13), following the manufacturer’s instructions (Bio-Rad Laboratories, Hercules, CA, USA). Data was read with the Bio-Plex Systems 100 (Bio-Rad Laboratories, Hercules, CA, USA).

### 2.18. Lung Histopathological Analysis

The left lungs were removed after inflation of both lungs with formalin at 20 cm H_2_O, embedded in paraffin blocks, cut into 4-μm sections and stained with hematoxylin and eosin. The peribronchial and perivascular inflammation was scored according to Barends et al. [17] as absent (0), minimal (1), slight (2), moderate (3), marked (4) or severe (5). Slide analysis was performed in a blinded manner.

### 2.19. Lung Tissue Immunofluorescence

Slides were deparaffinised through xylenes and an alcohol gradient and taken to water. Antigen retrieval was performed using H.I.E.R. (Heat Induced Epitope Retrieval) with Tris-EDTA buffer (pH 9). Non-specific antibody binding was blocked using protein block (Agilent Technologies, Santa Clara, CA, USA, cat X0909) for 10 min at room temperature. Sections were then incubated in a primary antibody cocktail consisting of: Goat anti-RSV (Abnova, Taipei City, Taipei, Taiwan, cat PAB13816) diluted to 1:200 and Rabbit anti-RFP (Abcam, Cambridge, UK, cat ab62341) diluted 1:600 in antibody diluent (Agilent, cat S3022) and incubated overnight at 4 °C. After washes in TBS-T, sections were incubated in a secondary antibody cocktail of: Donkey anti-Goat Alexaflour 488 (1:200) and Donkey anti-Rabbit Cy3 (1:300) and incubated for 1 h at room temperature. After washing in TBS-T, sections were incubated with DAPI to stain nuclei. Tissue autofluorescence was quenched in Sudan Black B solution for 45 min, washed and cover slipped using Vibrance mounting media (MJS Biolynx Inc., Brockville, ON, Canada). Whole slide scans were acquired on the Olympus Vs-120 scanner at 20× magnification.

### 2.20. Determination of Lung Viral Titres

To quantify RSV titres in the lungs of infected mice, the lungs were aseptically removed, weighed and homogenized using Tissue Ruptor (Qiagen). Serially diluted lung homogenate supernatants were inoculated onto HEp-2 cell monolayers followed by a 1% (*w/v*) agarose plaque assay. Lysis plate titration was performed using 0.05% neutral red and viral titre was expressed as plaque forming units per gram of lung (PFU/g of lung), as previously described [12].

### 2.21. Statistical Analysis

Data were presented as mean ±SEM. All in vitro experiments were performed in triplicate and repeated at least two times. In vivo experiments were performed at least two times (*n* ≥ 3 mice per group in each experiment). The results obtained were analyzed using GraphPad Prism 6 statistical software package. Comparisons between multiple groups were analyzed with one-way ANOVA and a posthoc Tukey or Bonferroni test. When appropriate, unpaired Student’s *t*-test or Mann–Whitney test were employed. The level of significance was set at *p* ≤ 0.05.

## 3. Results

### 3.1. ALMs Are Not Productively Infected by RSV and Are Resistant to RSV-Induced Cell Death

**Figure 1:**
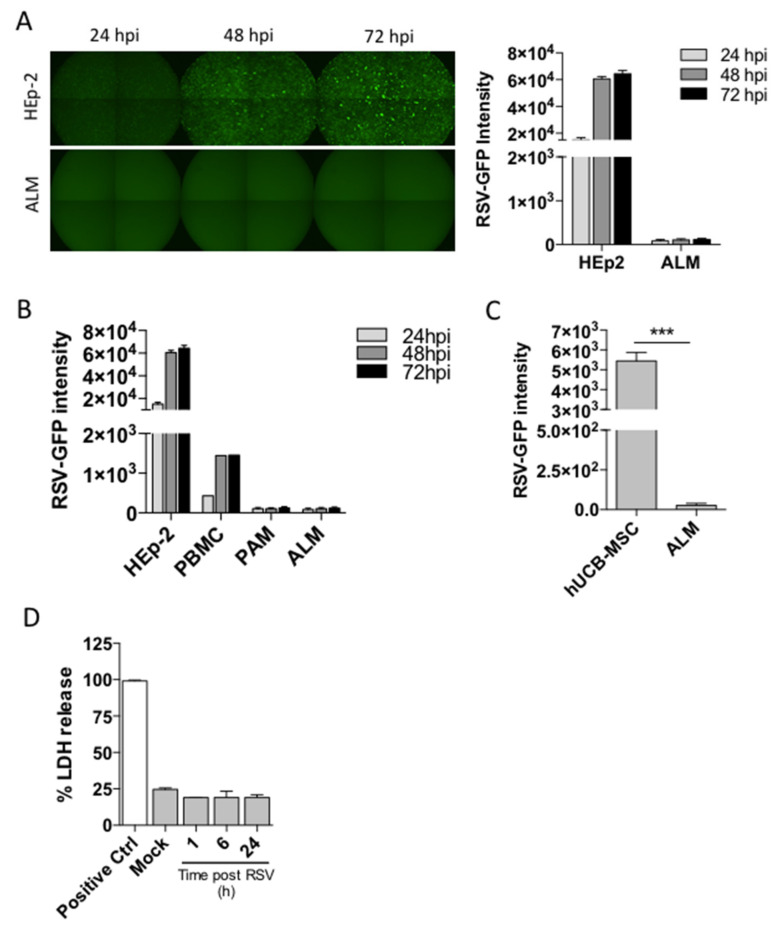
ALMs are not productively infected by RSV and are resistant to RSV-induced cell death. ALMs (2 × 10^5^/well) or HEp-2 cells (6 × 10^3^/well) were exposed to RSV-GFP at an MOI of 1. (**A**) GFP fluorescence was visualized by fluorescence microscopy (left panel) and quantified (right panel) at 24 h intervals for 72 h. (**B**) HEp-2 cells (6 × 10^3^/well), human peripheral blood mononuclear cells (2 × 10^5^/well), primary alveolar macrophages (2 × 10^5^/well) and ALMs (2 × 10^5^/well) were exposed to RSV-GFP and productive infection was quantified by fluorescence at 24 h intervals for 72 h. (**C**) Umbilical cord mesenchymal stromal cells (2 × 10^5^/well) or ALMs (2 × 10^5^/well) were exposed to RSV-GFP for 72 h and productive infection was quantified by fluorescence. (**D**) ALMs (3 × 10^5^/well) were exposed to RSV (MOI 1) for 1, 6, or 24 h. Afterwards, cell death was assessed by LDH release in ALMs supernatants. Data are representative of 2 independent experiments performed in triplicates and represent mean ±SEM. Data were analyzed with unpaired Student’s *t*-test. *** *p* = 0.0002.

Although airway epithelial cells constitute the primary target for RSV infection, RSV has been shown to infect alveolar macrophages ex vivo [18]. Thus, we sought to determine whether our stem cell-derived alveolar-like macrophages (ALMs) could be productively infected by RSV. ALMs or control HEp-2 cells were exposed to RSV-GFP for 24, 48 or 72 h. We imaged cells for GFP fluorescence, an indicator of active viral replication, and observed that HEp-2 cells displayed a fluorescence intensity of 1.51 × 10^4^ ± 1.6 × 10^3^ after 24 h of infection. Remarkably, ALMs did not show any fluorescence after 24 h of infection. There was a 4-fold increase in HEp-2 cell fluorescence intensity after 72 h of infection, whereas there was no change in ALM fluorescence intensity over the course of 72 h. (Figure 1A). We also evaluated the infection potential of other cell types including human peripheral blood mononuclear cells (PBMCs), primary mouse alveolar macrophages (PAMs) and human umbilical cord blood-mesenchymal stromal cells (hUCB-MSC). Our results show that PBMCs were productively infected by RSV although to a lesser extent when compared to HEp-2 cells, whereas PAMs and ALMs were not infected over the course of 72 h (Figure 1B). Interestingly, hUCB-MSCs were robustly infected after 72 h when compared to ALMs (Figure 1C). We then investigated whether RSV would trigger ALM death as we have previously described for primary AMs [19] by measuring lactate dehydrogenase (LDH) release. ALMs were resistant to RSV-induced lytic cell death over the course of 24 h (Figure 1D). Taken together, these data indicate that ALMs are not productively infected by RSV and are protected from RSV-triggered cell death.

### 3.2. ALM Exposure to RSV Confers a Time and Concentration Dependent Reduction in Viral Infectivity

**Figure 2:**
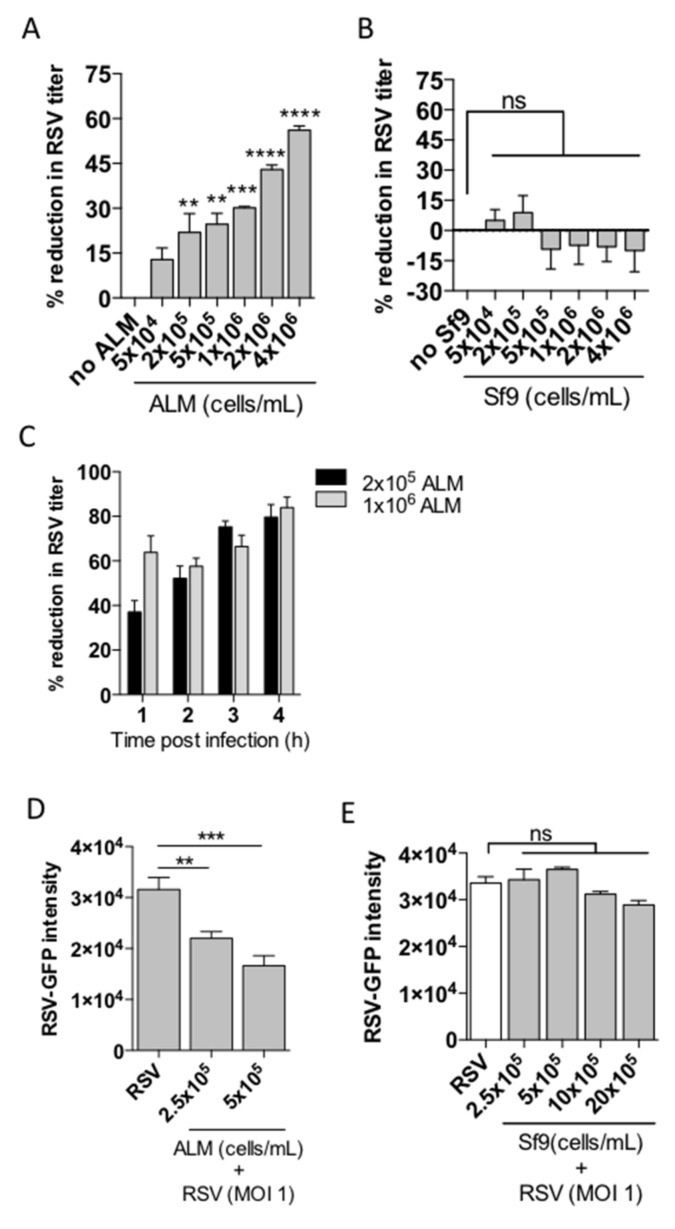
ALMs reduce RSV infectivity. Increasing numbers of (**A**) ALMs or (**B**) Sf9 cells were incubated with RSV (MOI 1) for 4 h at 37 °C under 5% CO_2_. Afterwards, the viral titre in the ALMs supernatants was assessed by plaque assay and the percent viral reduction in comparison to controls (no ALM) was calculated. (**C**) ALMs at the indicated numbers were incubated with RSV for 1–4 h and the reduction in viral infectivity was measured over time. (**D**) HEp-2 cells (6 × 10^3^/well) were seeded in black, flat, clear-bottom 96-well plates and allowed to attach overnight at 37 °C. The next day, ALMs (2.5 × 10^5^–5 × 10^5^/mL) and RSV (MOI 1) were added to the cultures for 48 h. GFP fluorescence was quantified as a readout of RSV replication. (**E**) HEp-2 cells (6 × 10^3^/well) were seeded in black 96-well plates overnight at 37 °C. The next day, Sf9 cells (2.5 × 10^5^–20 × 10^5^/mL) and RSV (MOI 1) were added to the cultures for 48 h. Afterwards, GFP fluorescence was quantified. Data are representative of 2 independent experiments performed in triplicates and represent mean ±SEM. Data were analyzed with one-way ANOVA with Tukey’s post hoc test. ** *p* < 0.01; *** *p* < 0.001; **** *p* < 0.0001. ns = not significant.

After determining that ALMs are not susceptible to productive RSV infection over the course of 72 h, we sought to understand more about the nature of this interaction between ALMs and RSV. We performed a series of experiments using a direct co-culture interaction of RSV with ALMs, or a control cell line, Sf9. We chose Sf9 cells as they do not express nucleolin, a putative receptor for RSV and we have previously shown that these cells are not infected by RSV [12]. We co-incubated RSV for 4 h at an MOI of 1 with increasing numbers of ALMs ranging from 5 × 10^4^ to 4 × 10^6^ ALMs. We measured the infectivity of the remaining RSV obtained from the co-incubation supernatants and found that as the number of ALMs increased the amount of infectious virus decreased (Figure 2A). Under the same experimental design, Sf9 insect cells ranging in number from 5 × 10^4^ to 4 × 10^6^ did not confer any significant reduction in RSV titre (Figure 2B). We then evaluated the temporal variability of this viral inhibition over the course of 4 h. At 2 × 10^5^ ALMs, the percent reduction in RSV titre ranged from 37.1% ± 5.1 at 1 hpi to 79.6% ± 5.6 at 4 hpi, whereas at 1 × 10^6^ ALMs, we noted an immediate and sustained reduction in viral titre ranging from 63.7% ± 7.4 at 1 hpi to 83.9% ± 4.7 at 4 hpi (Figure 2C). Next, we sought to determine whether the co-incubation of ALMs and RSV together with HEp-2 cells would inhibit the infection of HEp-2 cells in the context of direct co-culture. The HEp-2 cells were plated and cultured overnight at 37 °C to promote adherence. Then, ALMs were seeded onto the HEp-2 cells at a defined cell number (2.5 × 10^5^–5 × 10^5^) and RSV-GFP was inoculated into the co-culture environment at an MOI of 1 (respective to HEp-2 cell numbers). After 48 hpi the GFP signal in HEp-2 cells was quantified as a surrogate of RSV infectivity. Our results show that ALMs were able to significantly inhibit HEp-2 cell infection by RSV in the context of direct co-culture (Figure 2D). Conversely, when the experiment was performed with insect Sf9 cells, no significant change in viral infection was observed (Figure 2E). Taken together, these data suggest that ALMs interfere with RSV infection of epithelial cells.

### 3.3. ALMs Phagocytose and Inactivate RSV upon Viral Challenge

**Figure 3:**
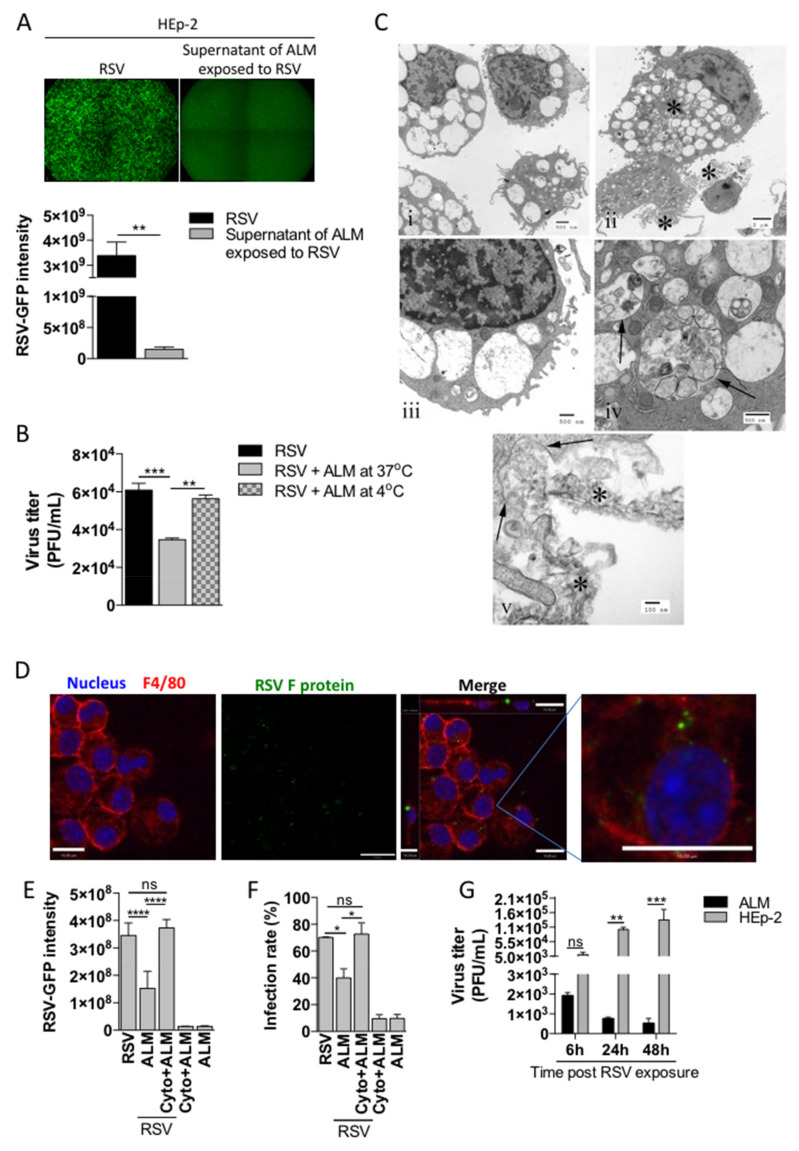
ALMs phagocytose RSV. (**A**) ALMs (1 × 10^6^/mL) were incubated with RSV-GFP (MOI 1) for 1 h at 37 °C with 5% CO_2_. Afterwards, the supernatants were collected and used to infect HEp-2 cells for 48 h. As a control, HEp-2 cells were directly infected with RSV-GFP (MOI 1) for 48 h. GFP fluorescence (top) was used to visualize RSV replication and GFP intensity was quantified (bottom) as a readout of RSV replication in HEp-2 cells. (**B**) ALMs (1 × 10^6^/mL) were incubated with RSV-GFP (MOI 1) for 1 h at either 37 °C or 4 °C. Afterwards, the supernatants were collected and used to infect HEp-2 cells for 48 h. After this period, RSV titre was measured in HEp-2 cells by plaque assay. (**C**) Electron micrographs of ALMs exposed to active RSV: (i) Unexposed macrophages. Note the limited phagocytosed material. Scale bar = 0.5 µm. (ii) RSV-exposed macrophages. Asterisks indicate accumulations of degraded RSV in both phagosomes and in close proximity to the plasma membrane. Scale bar = 2 µm. (iii). Unexposed macrophage. Note that the phagocytic vacuoles contain limited material when compared to the infected cells. Scale bar = 0.5 µm. (iv) RSV-exposed macrophage. Phagosomes (arrows) contain cellular and viral debris. Scale bar = 0.5 µm. (v) RSV-exposed macrophage. Ingestion through phagocytosis. Phagosomal membranes are seen ingesting degraded viral material (arrows). Degraded virus is seen in the extracellular space in close proximity to the cell surface. Scale bar = 0.1 µm. (**D**) ALMs (1 × 10^6^/mL) were exposed to RSV (MOI 1) for 4 h at 37 °C on coverslips. Afterwards, cells were fixed with 4% PFA and stained with Hoechst 33342 (1:4000), anti-F4/80 (1:100) and anti-RSV F protein (1:250) antibodies. Overlay of the fluorescence images are shown in the penultimate panel. The last panel shows the merged image magnified 4 times. Images are representative of 2 independent experiments. Images were taken with a Leica DMi8 confocal microscope. Scale bars = 15 μm. (**E**,**F**) ALMs (1 × 10^6^/mL) were pretreated with the actin polymerization inhibitor Cytochalasin D (Cyto–10 μM) for 1 h at 37 °C. Afterwards, ALMs were incubated with RSV-GFP (MOI 1) for 1 h. Then, ALMs were pelleted and the supernatants were added to HEp-2 cells for 48 h. After this period, (**E**) GFP intensity was quantified and (**F**) the infection rate was measured in HEp-2 cells. (**G**) ALMs and HEp-2 cells were exposed to RSV-GFP (MOI 1) for 6, 24 or 48 h. Afterwards, cells were harvested and lysed. The intracellular content was subjected to plaque assay to measure viral titre. Data are representative of 2 independent experiments performed in triplicates and represent mean ±SEM. Data were analyzed with unpaired Student’s *t*-test (**A**), one-way ANOVA with Tukey’s post hoc test (**B**,**E**,**F**) or 2-way ANOVA (**G**). * *p* < 0.05; ** *p* < 0.01; *** *p* < 0.001; **** *p* < 0.0001. ns = not significant.

Following the observed reduction in viral infectivity of RSV-ALM coincubation supernatants, we sought to determine potential mechanisms to explain this phenomenon. Macrophages have long been considered ‘professional’ phagocytes [20,21]—that is, a primary function is to engulf and destroy potentially pathogenic material, including viruses. We reasoned that phagocytosis may explain the observed antiviral effect of ALMs when they engaged with active RSV. We co-incubated ALMs with RSV-GFP for 1 h, then centrifuged the cells out of suspension. The supernatant was added directly to HEp-2 cells and after 48 h cells were examined by fluorescence microscopy. These data indicate that the supernatants of ALMs co-incubated with RSV-GFP contain significantly less RSV when compared to HEp-2 cells directly infected by RSV-GFP (Figure 3A). As phagocytosis is inhibited at low temperatures, we analyzed the infectivity of supernatants of ALMs co-incubated with RSV at 4 °C and found that RSV infectivity returned to RSV alone control levels (Figure 3B). This result supports the hypothesis that ALMs are phagocytosing RSV. We next examined the ALM cell pellet using transmission electron microscopy (TEM). TEM revealed viral-like particles associated with phagosome-like structures within ALMs (Figure 3C). Specifically, these structures were present in naïve ALMs, but remained mostly clear of internal material (Figure 3C(i,iii)); whereas RSV-exposed ALMs displayed substantial material contained within the phagosome-like structures (Figure 3C(ii,iv,v). In Figure 3C(v), phagosomal membranes (arrows) can be seen surrounding internalized viral material (asterisk). Immunofluorescence staining with antibodies against macrophage surface membrane protein F4/80 and the RSV fusion (F) protein was used to further examine RSV location. Confocal microscopy Z-stacks indicated viral proteins located both at the surface and in the cytoplasm of ALMs (Figure 3D). Finally, we studied the impact of the actin polymerization inhibitor cytochalasin D on phagocytosis. Treatment of ALMs with cytochalasin D abrogated the observed reduction in HEp-2 RSV infection (Figure 3E,F). Finally, we evaluated whether RSV particles internalized by ALMs or HEp-2 cells for different time points would be able to productively infect HEp-2 cells. ALMs or HEp-2 cells were exposed to RSV for 6, 24 or 48 h, cells were then lysed and the intracellular content was subjected to plaque assay. The resultant RSV titer decreased over the course of 48 h when RSV was internalized by ALMs, whereas RSV titers significantly increased over 48 h from HEp-2 cells (Figure 3G), suggesting that RSV particles internalized by HEp-2 cells remain viable while RSV internalized by ALMs are inactivated. Taken together, these data indicate that the antiviral effect of ALMs is mediated, in part, by phagocytosis and that RSV is inactivated inside ALMs.

### 3.4. ALMs Produce a Secreted Antiviral Factor Distinct from IFN-β

**Figure 4:**
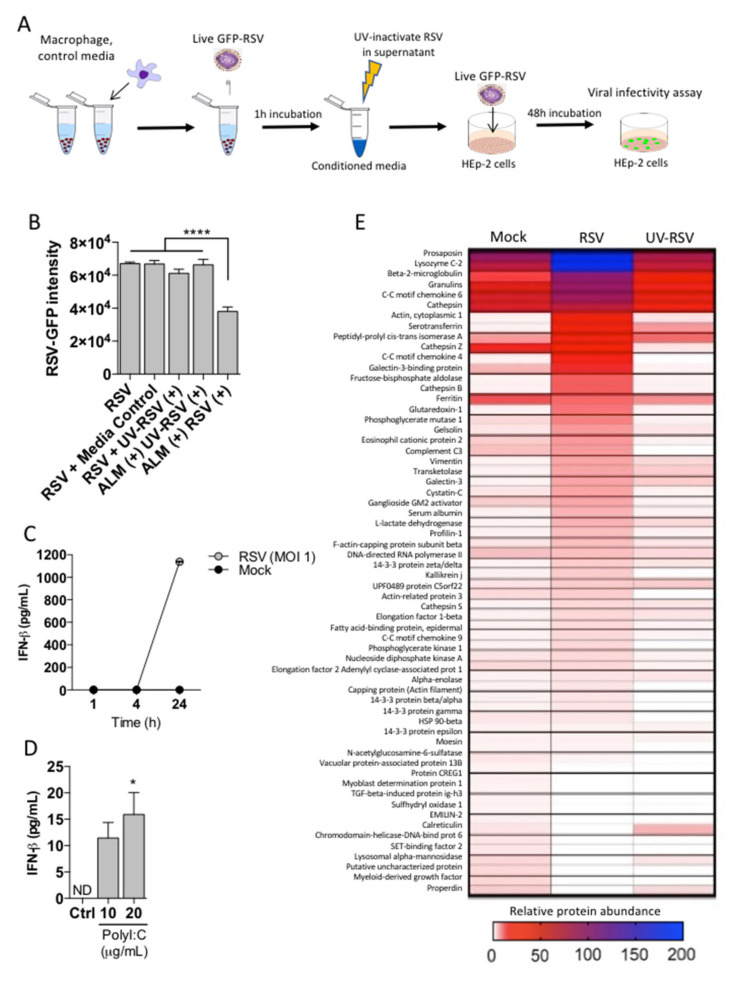
ALMs confer antiviral effects through a secreted factor. (**A**) Technical schematic indicating the work flow to measure secreted factor in ALMs supernatants. (**B**) ALMs (1 × 10^6^/mL) were exposed to RSV or UV-RSV at an MOI of 1 for 1 h. Afterwards, the supernatants were collected and UV-irradiated to neutralize residual active RSV. This was used as a conditioned media, which was then added onto previously seeded HEp-2 cells with competent RSV-GFP (MOI 1) and RSV replication was measured by GFP fluorescence. Control indicating HEp-2 cells with EMEM, media control indicates UV-irradiated EMEM, UV-RSV (+) indicates media containing UV-inactivated RSV, ALM (+) UV-RSV (+) indicates conditioned media from ALMs exposed to UV inactivated RSV, ALM (+) RSV (+) indicated conditioned media from ALMs exposed to RSV. (**C**,**D**) (**C**) ALMs (1 × 10^6^/mL) were stimulated with RSV (MOI 1) or mock-stimulated for 1, 4 or 24 h at 37 °C under 5% CO_2_. (**D**) ALMs were stimulated with TLR3 ligand Poly I:C (10 and 20 μg/mL) for 24 h at 37 °C under 5% CO_2_. Afterwards, supernatants were collected and IFN-β concentrations were determined using ELISA. (**E**) ALMs (1 × 10^6^/mL) were exposed to mock, RSV or UV-RSV at an MOI of 1 for 4 h. Afterwards, the supernatants were collected, concentrated and prepared for proteomic analysis as described in the Section 2. Data are representative of 2 independent experiments performed in triplicates and represent mean ±SEM. Data were analyzed with one-way ANOVA with Tukey’s post hoc test (**B**,**D**). * *p* < 0.05; **** *p* < 0.0001.

The possibility that ALMs secrete an antiviral factor was also considered, since ALMs have been shown to produce soluble factors under basal culture conditions [11]. To investigate the potential of a secreted factor that contributes to the anti-RSV effect of ALMs, a conditioned media assay was carried out. ALMs were exposed to active RSV, or UV-inactivated RSV (UV-RSV) for 1 h. Cells were pelleted and conditioned media collected and UV-irradiated to neutralize any residual infectious RSV. Conditioned media was added to HEp-2 cells simultaneously with competent active RSV-GFP and RSV infection was measured by fluorescence intensity 48 hpi (Figure 4A). HEp-2 cells were robustly infected by 48 h after introduction of conditioned media from the positive control (no ALMs) (Figure 4B, RSV). To control for potential changes in media proteins following UV irradiation, HEp-2 cells were incubated with UV-irradiated media and no change in RSV infectivity was observed (Figure 4B, media control). Media containing UV-irradiated RSV was included as an additional control and showed that presence of UV-RSV did not confer a change in RSV infectivity (Figure 4B, UV-RSV). Conditioned media from ALMs challenged with UV-RSV did not confer protection against viral infectivity in HEp-2 cells (Figure 4B, ALM + UV-RSV). Finally, conditioned media from ALMs incubated with live RSV conferred a significant reduction in RSV infection (Figure 4B, ALM + RSV). These data suggest an antiviral factor is present in ALM conditioned media. As type I interferons are commonly implicated in antiviral defense [22], we measured IFN-β in supernatants of ALMs exposed to active RSV for 1, 4, or 24 h (Figure 4C). IFN-β was not detectable over the first 4 h of ALM exposure to RSV during which antiviral activity was observed (as indicated in Figure 4B); though IFN-β was detected after 24 h (Figure 4C). To determine if ALMs respond to virus using TLR3, we measured the levels of IFN-β when ALMs were stimulated with the TLR3 agonist poly I:C. ALMs displayed a minimal response to TLR3 agonist poly I:C in a dose-dependent manner (Figure 4D). We then performed a proteomic analysis to better characterize the presence of IFN-β in the supernatants of ALMs exposed to mock, active RSV or UV-RSV. Our proteomics data show that ALMs exposed to RSV secreted a wide range of proteins, with prosaposin, lysozyme C-2, β2-microglobulin, granulins, CC-motif chemokine 6 (CCL6), cathepsin, serotransferrin, CC-motif chemokine 4 (CCL4), galectin-3-binding protein and cathepsin B among the top 10 most secreted proteins. However, ALMs incubated with RSV did not secrete IFN-β or any type of IFN for that matter (Figure 4E). Taken together, these data suggest that ALMs may secrete factors to inhibit RSV infection of epithelial cells; however, IFN-β is not one of these factors.

### 3.5. ALMs Prophylactically Mitigate the Effects of RSV Infection in Mice

**Figure 5:**
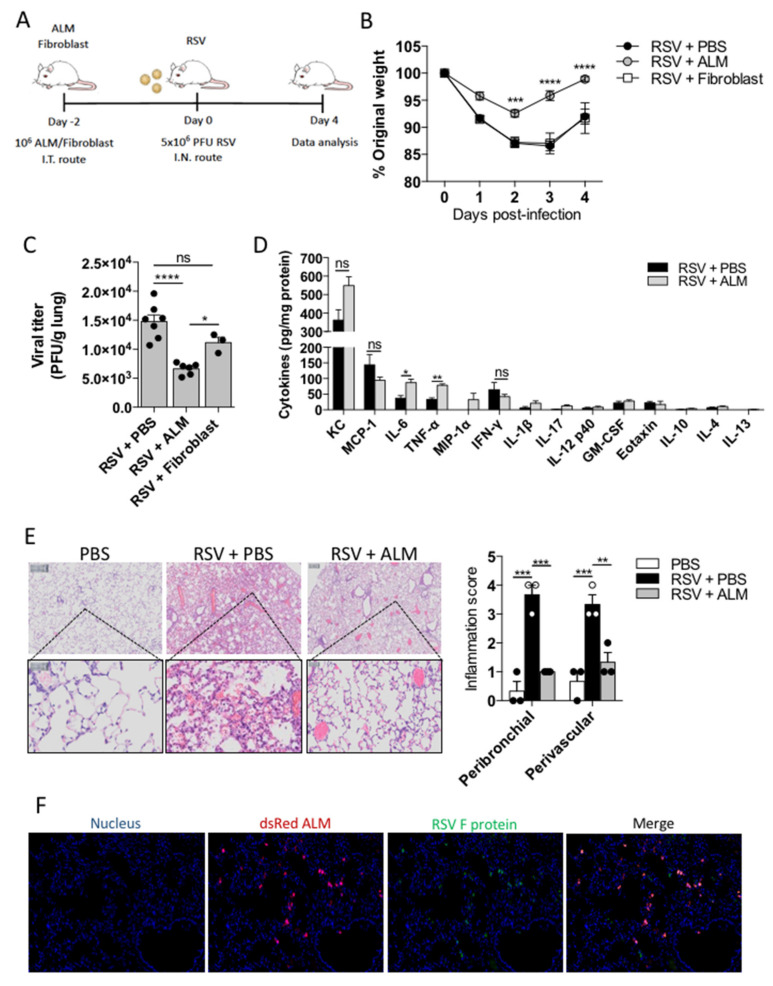
ALMs protect mice from RSV infection. (**A**) On day -2, BALB/c mice received an intratracheal injection with 1 × 10^6^ ALMs or 1 × 10^6^ fibroblasts as an inert cellular control. On day 0, mice were intranasally infected with RSV (5 × 10^6^ PFU). Analysis were performed on day 4 post-infection. (**B**) Percent of weight loss post-infection relative to initial body weight (day 0) (*n* = 8). (**C**) RSV viral titres obtained from lung homogenates measured by plaque assay (PFU/g of lung tissue) (*n* = 7 for RSV and RSV + ALM groups and *n* = 3 for RSV + fibroblast group). (**D**) Cytokines and chemokines were measured in the lung BAL of mice (*n* = 3) by multiplex Luminex^®^ technology and expressed as pg/mg protein. (**E**) Representative hematoxylin and eosin (H&E)-stained lung tissue images with their respective peribronchial and perivascular inflammation scores for each experimental group. (**F**) Immunofluorescence of lung tissue samples obtained from animals infected with RSV and treated with ALMs stained to identify dsRed-expressing ALMs and GFP- RSV. Data are representative of 2 independent experiments and are expressed as mean ±SEM. Data were analyzed with two-way ANOVA with Tukey’s multiple comparisons test (**B**,**C**,**E**) or with unpaired Student’s *t*-test (**D**). * *p* < 0.05; ** *p* < 0.01; *** *p* < 0.001;**** *p* < 0.0001. ns = not significant.

After establishing that ALMs can reduce RSV infectivity by phagocytosis in vitro, we sought to determine if ALMs would have any effect in vivo. We employed a prophylactic model of intervention by delivering 1 × 10^6^ ALMs or inert fibroblasts (cellular control) or PBS intratracheally to healthy mice 2 days prior to intranasal inoculation of 5 × 10^6^ PFU RSV (Figure 5A). Mice receiving fibroblasts or PBS in advance of RSV infection, lost weight in a virtually identical pattern with a peak weight loss at 3 days post infection (Figure 5B). Conversely, mice that had received ALMs displayed significantly less weight loss from day 2–4 post infection (Figure 5B). Moreover, animals receiving ALMs had a significantly lower viral titre in their lung tissue in comparison to PBS and fibroblast controls (Figure 5C). While infected mice that were treated prophylactically with ALMs showed an increase in the production of the pro-inflammatory cytokines IL-6 and TNF-α in their BAL fluid when compared to mice that did not receive ALMs (Figure 5D), the secretion of other cytokines and chemokines, including KC and MCP-1, was not significantly altered between the groups (Figure 5D). Hematoxylin and eosin staining showed less inflammation and cellular infiltration in RSV-exposed animals that were pretreated with ALMs in comparison to those that had not received any ALMs (Figure 5E). Specifically, there was a significant decrease in both peribronchial and perivascular inflammation scores presented by ALMs-treated infected mice compared to mice treated with PBS (Figure 5E). Furthermore, in infected mice, RSV and ALMs co-localized in the lung tissue (Figure 5F). Taken together, these data suggest that ALMs exhibit a prophylactic antiviral effect in vivo.

## 4. Discussion

Alveolar macrophages (AMs) are the tissue macrophages of the distal airways and are key cells in both health and disease [5]. In our original study describing the generation of ALMs, we suggested that these functional immune cells could be useful to address various pulmonary diseases by bolstering innate immunity [11]. We also demonstrated that ALMs are able to engulf bacteria and promote repair in injured airways (5). Here, we extend the utility of ALMs by evaluating these cells in a respiratory viral infection. We examined their role during a respiratory syncytial virus (RSV) infection, a common respiratory virus associated with significant morbidity and mortality and currently without any specific therapy. Initially, we analyzed whether ALMs could be productively infected by RSV. We found that, contrary to HEp-2 cells and PBMCs, ALMs could not be infected by this virus. In contrast to previous studies, our data show that PAMs are not susceptible to RSV infection [18,23,24]. However, there is a lack of evidence in the literature showing whether RSV infection of AMs is productive or abortive. Interestingly, RSV was able to infect hUCB-MSCs after 72 h, but not ALMs. MSCs have been previously shown to be infected by RSV in vitro and infective virus progeny are produced by MSCs [25]. Likewise, bone marrow-derived MSCs are permissive to influenza A H5N1 virus infection, losing viability and immunoregulatory activities [26]. The fact that ALMs are not susceptible to RSV infection allows for these cells to be considered as candidates for a broader cell-based antiviral therapy. In fact, the co-incubation of ALMs with RSV profoundly decreased the amount of infectious virus as measured by RSV titres. Furthermore, ALMs protected HEp-2 cells from RSV infection in a co-culture setting. Additionally, contrary to primary mouse AMs, which are susceptible to necroptosis triggered by RSV infection [19], ALMs were resistant to RSV-induced lytic cell death. Therefore, ALMs are resistant to both RSV infection and RSV-promoted cell death. However, we cannot exclude the possibility that the protocol used to generate ALMs may ‘pre-activate’ them, allowing them to outperform resting mouse AMs.

It has been reported that the co-culture of MSCs with H5N1 virus-infected alveolar epithelial cells reduces the adverse effect of infection on alveolar fluid clearance and protein permeability [27]. However, the effect of MSCs in preventing viral infection of respiratory epithelial cells is yet to be demonstrated–especially given in our current work, RSV productively infects MSCs [25]. Our data indicate that ALMs prevent HEp-2 cell infection by decreasing the amount of free infectious RSV particles. Thus, we hypothesized that phagocytosis could play a role in ALMs antiviral effect. Consistent with this assumption, we have previously shown that ALMs are able to phagocytose bacteria and apoptotic neutrophils [11]. We now extended these findings and show using two different techniques, transmission electron microscopy and immunofluorescence that ALMs phagocytose RSV. TEM revealed viral-like particles associated with phagosome-like structures within ALMs and the analysis of confocal microscopy Z-stacks indicated that RSV F protein was located both at the surface and in the cytoplasm of ALMs. Importantly, ALMs treated with cytochalasin D, which inhibits phagocytosis [28], did not reduce the ability of RSV to infect HEp-2 cells. This is consistent with previous data demonstrating macrophage phagocytosis of foot-and-mouth disease virus [29]. Interestingly, RSV can also be captured by eosinophils and have its infectivity significantly reduced, though in the setting of eosinophils, RSV was not surrounded by a phagosome-like membrane [30].

Type I interferons (IFNs) are induced by viral infections [22] and AM-derived type I IFNs orchestrate innate immunity against RSV infection [31]. Thus, we examined if a soluble factor released by ALMs in response to RSV would be involved in limiting viral infectivity. Analyzing the conditioned media obtained from ALMs exposed to active RSV, we found that this media promoted a significant reduction in RSV infectivity. The proteomics data from ALM conditioned media revealed that ALMs incubated with RSV secrete a wide range of proteins, including lysozyme, β2-microglobulin, and chemokines such as CCL6 and CCL4. Lysozyme secretion has been shown to be inhibited by influenza virus, which may predispose to a severe secondary bacterial infection following a viral infection [32]. Therefore, the fact that ALMs secrete lysozyme in response to RSV might be beneficial to the host. On the other hand, the increased secretion of β2-microglobulin may be detrimental to the host as previous studies have associated elevated levels of β2-microglobulin with disease severity in different viral infections, including influenza and COVID-19 [33,34]. Corroborating with our data, the chemokines CCL6 and CCL4 (also known as MIP-1β) have been previously reported to be upregulated by alveolar macrophages after RSV infection in vitro and by airway cells following RSV infection in vivo [35,36]. Nevertheless, in our experiments, IFN-β was unlikely to be responsible for the reduction in RSV infectivity, since IFN-β was not secreted by ALMs in the first 4 h after viral exposure. In accordance, AMs have been shown to control RSV replication in the absence of type I IFNs production or signaling [37], indicating that these cells are endowed with mechanisms different from the canonical IFN pathway to restrict RSV infection. Therefore, it is possible that AMs and ALMs share the ability of limiting RSV infection independently of type I IFN. Importantly, the identification of the soluble factor mediating the antiviral effect of ALMs requires further investigation.

Due to the high infectivity of RSV, almost 70% of all children are infected with the virus in the first year of life, and by age 3, almost 100% of children have experienced at least one infection with this virus [38,39]. Despite substantial research efforts, there is yet no effective RSV vaccine available. Palivizumab, a monoclonal antibody against the RSV Fusion protein, has been shown to reduce the severity of infection in babies; however, palivizumab is expensive and thus administration is limited to high-risk groups, such as preterm infants and those suffering from cardiovascular diseases, bronchopulmonary dysplasia or immunosuppression [40]. Therefore, we sought to investigate whether ALMs would have a protective effect in vivo and could work as a prophylactic antiviral therapy. We delivered ALMs intratracheally 2 days in advance of intranasal RSV inoculation. Our data clearly demonstrate that mice receiving ALMs were protected from RSV-induced weight loss, which was corroborated by the significant reduction in lung viral titres. Notably, the prophylactic treatment with ALMs profoundly reduced lung inflammation as evidenced by peribronchial and perivascular inflammation scores. Interestingly, mice that received ALMs exhibited an increase in TNF-α and IL-6 secretion in their BAL fluid, which might have potentiated ALMs antiviral effect, as these cytokines have been previously shown to exert a strong antiviral activity against respiratory viruses, including RSV and influenza [41,42]. Moreover, chemokines cooperate with TNF-α to provide an optimal antiviral immunity and to enhance inflammation [43]. Together with the decreased viral titre data, the co-localization of ALMs and RSV in the lung tissue strongly suggests that the virus is neutralized by ALMs following infection. Similarly, AMs seem to be essential for the early antiviral response against RSV infection [7,8,9] and to prevent lethal influenza virus pneumonia in mice [44]. On the other hand, the success of MSCs in the treatment or prevention of respiratory virus infections has been controversial. While earlier studies have found MSCs not to be protective against influenza H1N1-triggered lung injury in mice [45,46], more recent investigations have reported that the systemic delivery of MSCs significantly reduced virus-induced mortality, weight loss and lung inflammation in mouse models of influenza A infection [27,47,48]. Despite the suggestive evidence for a potential beneficial role of MSCs during respiratory viral infections, there are limited published clinical data available. In a recent pilot investigation from the Youan Hospital in Beijing, China, ACE2-deficient MSCs were intravenously administered to 7 patients with COVID-19 pneumonia. The patients were followed for 14 days after MSCs or placebo delivery. The authors found that, after MSCs treatment, peripheral lymphocyte numbers were increased, C-reactive protein levels decreased, the levels of the pro-inflammatory cytokine TNF-α were significantly reduced while those of the anti-inflammatory IL-10 were increased in the MSC group compared to the placebo group [49]. Thus, it seems that the administration of MSCs was safe and effective for COVID-19 pneumonia patients. Importantly, the fact that these MSCs were ACE2-deficient may have protected the cells from SARS-CoV-2 infection and allowed them to play an essential antiviral or anti-inflammatory role during COVID-19 pneumonia. In our current work, we demonstrate that ALMs are not susceptible to RSV infection. It is worth to mention that adding ALMs after infection would be a logical next step and an important one to evaluate the potential therapeutic application of ALMs during RSV infection. Additionally, future work will explore ALMs and other respiratory viruses in order to explore the use of ALMs as a broad-spectrum agent to prevent respiratory viral infection.

In summary, our data demonstrate that ALMs are not productively infected by RSV and prevent the infection of epithelial cells by decreasing the amount of free infectious virus. In addition, we found that ALMs displayed two different mechanisms for protecting epithelial cells from RSV infection: phagocytosis of RSV particles and the release of an antiviral secreted factor different from type I IFN. Furthermore, intratracheal administration of ALMs protected mice from virus-induced weight loss and decreased lung viral titres and inflammation after RSV exposure, indicating that ALMs can prevent the pathogenesis of RSV infection. Our results justify further exploration into the translational value of stem cell-derived alveolar-like macrophages as a novel cell-based therapy for pulmonary viral infections.
